# Study of circulating myeloid derived suppressor cells (MDSC) in patients with breast cancer undergoing neo-adjuvant chemotherapy; interim results

**DOI:** 10.1186/2051-1426-1-S1-P66

**Published:** 2013-11-07

**Authors:** Robert Wesolowski, Joseph Markowitz, Bonnie Paul, Sarah Carothers, Mahmoud Abdel-Rasoul, William E Carson

**Affiliations:** 1 Medical Oncology, The Ohio State University, Columbus, OH, USA; 2 Surgical Oncology, The Ohio State University, Columbus, OH, USA; 3 The Ohio State University Comprehensive Cancer Center, Columbus, OH, USA; 4 Center For Biostatistics, The Ohio State University, Columbus, OH, USA

## Introduction

Patients (pts) with breast cancer (BC) who achieve complete pathologic response (pCR) to neo-adjuvant chemotherapy (NAC) have better survival than pts without pCR. It is hypothesized that circulating levels of MDSC may be a potential predictive biomarker for NAC.

## Methods

Pts with operable BC electing to have NAC are eligible. Pts usually receive an anthracycline (AC) regimen followed by a taxane (T) (+ trastuzumab for HER-2/neu+ BC). Circulating levels of MDSC were measured by flow cytometry as a percentage of peripheral blood mononuclear cells prior to cycle 1, 2 of AC and cycle 1 and 4 of T. If any other NAC regimen is used, MDSC were measured prior to 1st, 2nd and last cycle. MDSC were identified as HLA-DR-, CD11b+, CD33+ cells with granulocytic (G-MDSC) and monocytic (M-MDSC) subsets expressing CD15 and CD14, respectively. The 1o objective is to study the changes in MDSC % in response to NAC. A sample size of 24 pts (6 with pCR and 18 without pCR) provides 80% power to detect at least an effect size of 1.5 standard deviation between the responders and non-responders using a 2 sided, 2 sample t-test with an α level of 0.05.

## Results

To date, 14 of 24 pts have been enrolled (stage I [N=1], stage II [N=13], triple negative (TN) [N=8], HER-2/neu+ [N=5], hormone receptor (HR)+ [N=1]). Median age is 46 (range 32-69). G-MDSC % and 95% confidence intervals [95% CI] were 1.45 [0.38-2.51], 7.59 [3.40-11.78], 11.76 [3.67-19.85], 3.17 [0 - 7.49] at time points 1-4 respectively. M-MDSC % was smaller but followed a similar trend. This trend was also seen in pts with TN and Her-2/neu+ BC but not in 1 pt with HR+ BC who had persistent increase in MDSC. Of 5 pts who completed NAC, 4 had pCR. We found that MDSC% initially increased during NAC but decreased at the end of treatment in pts with pCR (G-MDSC percentages [95% CI]: 0.27 [0-0.76], 9.32 [0.97-16.8], 9.31 [0.45-21.9], 1.22 [0.18-2.31]. Conversely, MDSC % continued to rise in the pt that did not have pCR (G-MDSC: 0.36, 3.37, 11.3 at time points 1-3, respectively). M-MDSC % followed the same trend in patients with or without pCR.

## Conclusion

This preliminary data suggests that G-MDSC % at the end of chemotherapy is low in patients with pCR but continues to rise in patients who do not respond to chemotherapy. More data is needed to confirm these results.

